# Frequency and Predictors of Diabetes Mellitus in Smear-Positive Pulmonary Tuberculosis Patients: A Cross-Sectional Study

**DOI:** 10.7759/cureus.75809

**Published:** 2024-12-16

**Authors:** Abdul Baqi, Kamran K Sumalani, Nousheen Akhter, Maqbool Ahmed, Khalid Ahmed, Dimple Chawla, Nadeem Rizvi

**Affiliations:** 1 Pulmonology, Shaikh Khalifa Bin Zayed Al-Nahyan Medical Complex, Quetta, PAK; 2 Pulmonology, Jinnah Postgraduate Medical Centre, Karachi, PAK; 3 Pulmonology, Bahria University Medical and Dental College, Karachi, PAK; 4 Pulmonology, Fatima Jinnah General and Chest Hospital, Quetta, PAK

**Keywords:** diabetes mellitus, mycobacterium, prevalence, sputum smear positive, tuberculosis

## Abstract

Introduction

Tuberculosis (TB) is an infectious disease caused by *Mycobacterium tuberculosis*. Various studies have established an association between diabetes mellitus (DM) and pulmonary TB. This study describes the prevalence of DM and its predictors in smear-positive TB patients.

Methods

This descriptive cross-sectional study was conducted at the Department of Chest Medicine at Jinnah Postgraduate Medical Centre (JPMC), Karachi, Pakistan, from December 2015 to June 2016. The inclusion criteria were as follows: drug-sensitive pulmonary TB patients of either gender, age ≥ 18 years, registered at the TB clinic JPMC, who consented to participate in the study. Pregnant/lactating patients, drug-resistant TB patients, and those who did not consent to participate in the study were excluded. Fasting blood sugar (FBS) and glycated hemoglobin (HbA1c) were done for all patients included in the study. Sociodemographic data, along with FBS, random blood sugar (RBS), and HbA1c, were entered in a pre-designed proforma. A sedentary lifestyle was recorded as a subjective measure of the proforma. Diabetes was diagnosed if FBS was ≥126 mg/dL or HbA1c was ≥6.5%.

Results

Out of 100 TB patients included in the study, 18 (18%) were diagnosed with diabetes. Patients who were smokers, had a sedentary lifestyle, and had a family history of diabetes had a significantly high prevalence of diabetes (p-value < 0.0001). Age, gender, and body mass index (BMI) did not influence the prevalence of diabetes in TB patients.

Conclusion

The study revealed an increased occurrence of diabetes among patients with smear-positive pulmonary TB. Diabetes is more common in TB patients who smoke, have a sedentary lifestyle, and those who have a family history of diabetes. A comprehensive management plan shall be in place to cater to TB and diabetes comorbidity, including effective management of TB and optimal glycemic control.

## Introduction

Tuberculosis (TB) is an infectious disease caused by *Mycobacterium tuberculosis* [1]. TB remains one of the main causes of morbidity and mortality in low- to middle-income countries, where the number of individuals with diabetes mellitus (DM) is rapidly increasing [2,3]. The burden of TB is alarmingly high in Pakistan. Pakistan ranks fifth in the list of 30 high-burden TB countries, with an estimated 518,000 TB cases, including 15,000 multidrug-resistant (MDR)-TB cases [4].

As far as diabetes is concerned, there are 33 million people living with type II DM, according to the most recent data. This makes it the third-largest diabetes population globally. Additionally, 11 million people in Pakistan have impaired glucose tolerance, and approximately 8.9 million people with diabetes are undiagnosed [5].

There is an association between DM and pulmonary TB [6]. Subclinical diabetes arises as a response to the stress caused by an existing infection, while the condition of glucose intolerance tends to improve or return to normal after successful anti-tuberculous treatment [7]. Likewise, diabetes poses a considerable risk, as evidence suggests that the emerging epidemic of diabetes is fanning the spread of TB. In diabetes, there is gross impairment in chemotaxis, phagocytosis, activation, and antigen presentation by alveolar macrophages, monocytes, and T-lymphocytes, and there is decreased phagocytosis by polymorphonuclear leukocytes as well. There is an impairment in cytokine production and the natural killing of pathogens; hence, overall cell-mediated immunity, which is vital in protection against *M. tuberculosis*, is grossly impaired in persons with diabetes [8-11].

The increasing burden of DM poses a threat to efforts towards ending TB [12]. Diabetes triples the risk of acquiring TB infection [13]. TB patients with DM have a higher risk of treatment failure, relapse, and an accelerated emergence of TB drug resistance [14]. TB patients with DM are almost four times as likely to experience a relapse of their TB after being cured, nearly twice as likely to develop MDR, and twice as likely to die during TB treatment, compared to TB patients without DM [13,15].

Information on the magnitude of the TB-diabetes co-epidemic is still scarce. This study aimed to determine the prevalence of DM and its associated factors among TB patients.

## Materials and methods

This descriptive cross-sectional study was conducted at the Department of Chest Medicine at Jinnah Postgraduate Medical Centre (JPMC), Karachi, Pakistan, from December 2015 to June 2016. The study received approval from the Institutional Review Board Committee of JPMC Karachi, approval no. 10040, dated December 22, 2015. The sample was collected via non-probability consecutive sampling. The inclusion criteria were as follows: drug-sensitive pulmonary TB patients of either gender, age ≥ 18 years, registered at the TB clinic JPMC, who consented to participate in the study. Pregnant/lactating patients, drug-resistant TB patients, and those who did not consent to participate in the study were excluded.

Fasting blood sugar (FBS) and glycated hemoglobin (HbA1c) were done for all patients included in the study. Sociodemographic and anthropometric data, along with FBS and HbA1c, were entered in a pre-designed proforma. A sedentary lifestyle was recorded as a subjective measure of the proforma. This includes questions regarding time spent sitting at a desk, visiting friends, cooking, reading, sitting or lying down to watch television, sitting or lying down to listen to the radio, sitting or lying down to use a mobile phone, etc. A sedentary lifestyle was subjectively recorded if there were more than six hours of inactivity during awake hours. Diabetes was diagnosed if FBS was ≥126 mg/dL or HbA1c was ≥6.5%.

Data were analyzed using IBM SPSS Statistics for Windows, Version 20 (Released 2011; IBM Corp., Armonk, NY, USA). Mean ± SD were calculated for age, duration of smear-positive pulmonary TB, and body mass index (BMI). Frequency and percentage were calculated for gender, DM, and the predictors, i.e., age, BMI groups, sedentary lifestyle, smoking status, and family history of diabetes (including parents, siblings, and first-degree relatives). A p-value of less than 0.05 was considered significant.

## Results

This study included a total of 100 patients. The mean age of the study participants was 42.56 ± 12.24 years. Males comprised 60 (60%). Details regarding the demographics and clinical characteristics of the study participants are shown in Table 1.

**Table 1:** Socio-demographics characteristics of enrolled patients. BMI: Body mass index; N: Frequency; SD: Standard deviation: TB: Tuberculosis

Parameters		Values
Age in years (mean ± SD)		42.56 ± 12.24
Gender (N, %)	Male	60, 60%
	Female	40, 40%
BMI in kg/m^2 ^(mean ± SD)		18.19 ± 2.46
Duration of smear-positive TB in months (mean ± SD)		5.26 ± 1.24
Diabetes mellitus (N, %)	Positive	18, 18%
	Negative	82, 82%
Sedentary lifestyle (N, %)		15, 15%
Smoker (N, %)		15, 15%
Family history of diabetes mellitus (N, %)		19, 19%

Out of a total of 100 patients, 18/100 (18%) were diagnosed with diabetes. Diabetes was more prevalent in TB patients who were smokers, had a sedentary lifestyle, and had a family history of diabetes (p-value < 0.0001). However, diabetes was found to be equally prevalent in both genders and all age groups. Likewise, BMI and duration of TB did not affect the prevalence of diabetes in TB patients. Statistical details of predictors of DM in TB patients are shown in Table 2.

**Table 2:** Stratification of diabetes mellitus with respect to its predictors in patients with TB. BMI: Body mass index; N: Frequency; TB: Tuberculosis

Variable		Diabetes mellitus		Chi-square value	p-value
		Yes (N, %)	No (N, %)		
Age in years	20-40	11 (18.03%)	50 (81.96%)	0.000	0.991
	>40	7 (17.94%)	32 (82.05%)		
Gender	Male	12 (20%)	48 (80%)	0.407	0.524
	Female	6 (15%)	34 (85%)		
Duration of smear positive in months	0-2	10 (14.28%)	60 (85.71%)	2.181	0.140
	>2	8 (26.66%)	22 (73.33%)		
BMI (kg/m^2^)	<18	13 (16.66%)	65 (83.33%)	0.427	0.513
	18-25	5 (22.72%)	17 (17.27%)		
Smoking	Yes	12 (80%)	3 (20%)	45.959	0.000
	No	6 (7.05%)	79 (92.94%)		
Sedentary lifestyle	Yes	11 (73.33%)	4 (26.66%)	36.607	0.000
	No	7 (8.23%)	78 (91.76%)		
Family history of diabetes	Yes	14 (73.68%)	5 (26.31%)	49.277	0.000
	No	4 (4.93%)	77 (95.06%)		

## Discussion

In our study, DM was present in 18/100 (18%) of patients with smear-positive pulmonary TB. Literature shows that the prevalence of DM among TB patients ranges from 5% to 41.1% [16-19]. An Indian study reported that almost half of TB patients are either diabetic or have pre-diabetes [18]. This wide variation may be due to differences in the settings where the studies were conducted. Other factors may include differences in the underlying prevalence of diabetes in the general population, the socio-economic characteristics of study participants, and the diagnostic approaches used.

The present study did not find an age difference in the diagnosis of diabetes. This is discordant with the results of studies conducted in India and other parts of the world, where older patients had a higher prevalence of diabetes [19]. This may be due to urbanization and increasing life expectancy. Also, diabetes is more common in the older age group in the general population, making individuals more prone to infections, including TB. We had younger patients in our study group, likely the reason that no significant difference could be demonstrated according to age. Similarly, there was no gender difference in the prevalence of diabetes. This was in concordance with the results of a study by Sharma et al. [20]. Although some studies found diabetes to be more common in males [21], others showed higher odds of getting diabetes among females compared to males [22]. This may be a good area for future research, exploring the variation in the prevalence of diabetes and DM-TB comorbidity between genders.

Diabetes was found to be equally prevalent across a range of BMIs. There was also no difference in diabetes prevalence according to the duration of TB diagnosis. One study reported that patients with dual comorbidity of DM and TB are underweight [22]. Another study reported a higher BMI in TB patients who also have diabetes, but no significant association was shown, probably due to an insufficient sample size [18].

We could not find any difference in the prevalence of diabetes in TB patients according to the duration of smear positivity, whether it was less than or more than two months. It is worth noting that TB may cause transient hyperglycemia, so the prevalence of diabetes may be miscalculated. To cater for this overestimation of DM, screening in TB patients may be more appropriate after TB treatment starts to be effective [22].

Diabetes was found to be significantly more prevalent in participants who smoked, had a sedentary lifestyle, or had a family history of diabetes. Smoking was shown to be associated with a high prevalence of diabetes in TB patients [23,24], while other studies did not show any association, likely due to underreporting of smoking habits by study participants [22]. Like our study, diabetes was shown to be more prevalent in patients with a family history of diabetes in studies conducted in Asia and Africa [23-25]. This may be due to a genetic predisposition to diabetes.

The limitations of our study are as follows: it is a single-center study, so the results may not be generalized. Secondly, self-reported use of tobacco may have led to over- or under-estimation. Thirdly, the study design was cross-sectional, so a causal association cannot be demonstrated for DM-TB comorbidity and associated predictors. Fourth, patients who were admitted with severe TB infection were not included in the study. Further research is needed to better understand the interplay between the occurrence of the two epidemics and their associated predictors. It may be recommended that robust guidelines be implemented to screen for DM in TB patients in countries where both diseases are endemic.

## Conclusions

The study concludes that DM is a common comorbidity in patients with TB. According to the present study, one in every five TB patients also had diabetes. Among all these patients with TB-DM comorbidity, more than three-quarters were smokers. A sedentary lifestyle and a family history of diabetes were also found to be more common in patients with TB-DM comorbidity.

Screening for diabetes in TB patients should be strengthened, and optimal care should be provided to these patients, ensuring both optimal TB treatment and glycemic control. Early diagnosis and management of DM may enhance TB control efforts. A sound understanding of the underlying mechanisms and predictors of the association between TB and DM may help design integrated treatment strategies by which the dual disease burden may be overcome.
